# AI in Adipose Imaging: Revolutionizing Visceral Adipose Tissue, Ectopic Fat, and Cardiovascular Risk Assessment

**DOI:** 10.1007/s11883-025-01356-1

**Published:** 2025-10-09

**Authors:** Sneha R. Kandi, Rohan Khera, Sanjay Rajagopalan, Ian J. Neeland

**Affiliations:** 1 Department of Medicine, Case Western Reserve University School of Medicine, Cleveland, OH USA; 2 University Hospitals Cleveland Medical Center, Cleveland, OH USA; 3 Section of Cardiovascular Medicine, Department of Internal Medicine, Yale School of Medicine, New Haven, CT USA; 4 Cardiovascular Data Science (CarDS) Lab, Yale School of Medicine, New Haven, CT USA; 5 Center for Outcomes Research and Evaluation, Yale-New Haven Hospital, New Haven, CT USA; 6 Section of Health Informatics, Department of Biostatistics, Yale School of Public Health, New Haven, CT USA; 7 Harrington Heart and Vascular Institute, University Hospitals, Cleveland, OH USA

**Keywords:** Visceral adipose tissue, Ectopic fat, Artificial intelligence, Deep learning, Cardiovascular risk, Medical image segmentation

## Abstract

**Purpose of Review:**

This review explores the role of artificial intelligence (AI) in visceral adipose tissue (VAT) and ectopic fat imaging. It aims to evaluate how AI may be used to enhance the efficiency and accuracy of cardiovascular disease (CVD) risk assessment. It addresses key questions regarding AI’s capabilities in risk prediction, segmentation, and integration with large volume data for CVD risk assessment.

**Recent Findings:**

Recent studies demonstrate that AI, powered by deep learning models, significantly improve VAT and ectopic fat segmentation. AI can also be used to facilitate early detection of cardiometabolic risks and allows integration of imaging with clinical data for a more personalized approach to medicine. Emerging applications include AI-enabled telehealth and continuous monitoring through wearable technologies.

**Summary:**

AI is transforming VAT and ectopic fat imaging by enabling more precise, personalized, and scalable assessments of fat distribution and cardiovascular risk. While challenges remain, such as model interpretability, future research will likely focus on refining algorithms and expanding AI’s clinical applications, potentially redefining obesity and CVD risk management.

## Introduction

Artificial Intelligence (AI) is reshaping the landscape of medical imaging by presenting exceptionally sophisticated tools for the assessment and quantification of adipose tissue [1]. AI models leverage large datasets of annotated medical images to develop the capability to detect subtle patterns in adipose distribution that may be indicative of metabolic disorders, cardiovascular risk, or other obesity-related health conditions [1]. AI powered radiomics enables the extraction of dimensional imaging features that go beyond conventional fat quantification, which offer deeper insights into adipose tissue composition and function [2]. These advancements are revolutionizing diagnostic capabilities and promoting personalized medicine by integrating adipose imaging with clinical risk prediction models [3]. As AI continues to evolve, its applications in adipose imaging are expanding as well. This will allow for clinicians to detect early predictors of metabolic dysfunction, improving patient stratification, and ultimately transforming the management of obesity and its related comorbidities [3].

### Metabolic Implications

Approximately 80% of total adipose tissue is subcutaneous (SCAT) and visceral adipose tissue (VAT) comprises 10–20% in men and 5–10% in women [4]. SCAT expands through adipocyte hyperplasia and once these adipocytes are saturated, excess fatty acids and triglycerides deposit in visceral fat and ectopic sites including the heart, liver, muscles, kidneys, and pancreas [5]. VAT holds significant clinical importance due to its strong correlation with cardiovascular diseases [6]. This importance is due to the fact that VAT is metabolically active, leading it to often be associated with conditions such as hypertension, diabetes, and atherosclerosis [6]. For similar reasons, VAT is also a key determinant in cardiovascular risk [7]. Unlike SCAT, VAT releases pro-inflammatory cytokines including interleukin-6 (IL-6) and tumor necrosis factor-alpha (TNF-α) which play a large role in systemic inflammation and insulin resistance [7]. The accumulation of VAT is linked to metabolic syndrome which is characterized by a constellation of obesity, hypertension, dyslipidemia and glucose intolerance [6]. Multiple studies have identified VAT is an independent predictor of major cardiovascular events such as myocardial infarction and stroke [8]. Beyond these metabolic effects, inflammatory mediators secreted from VAT are also implicated in vascular dysfunction, contributing to endothelial dysfunction, arterial stiffness, and increased atherogenesis [9]. Research indicates that VAT plays a role in regulating lipid metabolism, and it has been shown to promote an atherogenic lipid profile, often exacerbating cardiovascular risk [9]. Ectopic fat is also an important determinant of cardiovascular disease (CVD) and metabolic risk. Epicardial and pericardial fat, have been linked to increased risk for CVD and arrhythmias [10, 11]. Liver fat (hepatic steatosis) is strongly associated with type 2 diabetes and risk for metabolic-dysfunction associated steatotic liver disease (MASLD) and metabolic-dysfunction associated steatohepatitis (MASH) [11]. Fat infiltration in skeletal muscle is associated with obesity and a dysmetabolic state and has been shown to be modifiable with anti-obesity medications such as glucagon-like peptide 1 receptor agonists (GLP-1 RA) [11, 12]. These mechanisms highlight the need and necessity for AI given the role of accurately quantifying VAT and ectopic fat to improve CVD risk prediction models.

### Current Imaging Techniques

Traditional imaging techniques such as computed tomography (CT) and magnetic resonance imaging (MRI) have been the gold standards for VAT and ectopic fat quantification, however they are limited in that their manual analysis is time consuming and prone to variability [13, 14]. Current techniques for assessing VAT and ectopic fat can be classified into direct and indirect methods [15]. MRI and CT are direct imaging techniques that are considered the gold standard for VAT quantification. They both have high spatial resolution which allows them to differentiate VAT from SCAT with a high degree of accuracy [15]. MRI is an idea modality for repeated assessment since it provides exceptional soft tissue contrast without exposing patients to ionizing radiation. However, MRI is costly, time-consuming, and requires specialized equipment that may not be widely available in all clinical settings [16]. In contrast, CT offers prompt and reliable VAT quantification. CT is often sparingly used in routine screening or serial measurements in asymptomatic individuals due to efforts to limit patient’s exposure to ionizing radiation [14], . While both MRI and CT provide quality images, manual or semi-automated segmentation of VAT and ectopic fat remains a challenge. The processing of large volume data sets often requires significant expertise and time for accurate analysis [17].

Indirect methods for VAT assessment include dual-energy X-ray absorptiometry (DEXA), ultrasound (US), and plethysmography. Each of these modalities are associated with varying degrees of accuracy and accessibility. DEXA is most commonly used in clinical settings for assessing body composition and accurate estimates of total body fat, lean mass, and bone density [18]. While DEXA can differentiate between fat and muscle tissue, its ability to accurately distinguish VAT from SCAT is limited, making it a less precise alternative to MRI and CT [19]. Ultrasound, being relatively cheap and non-invasive, can also be used to estimate VAT thickness, and has been proven to be particularly useful in point-of-care settings [20]. Conversely, ultrasound’s accuracy is highly operator dependent, and its depth penetration is limited in obese individuals which affects the reliability of VAT measurements [20]. Plethysmography, which measures body volume and density using air displacement, provides an indirect assessment of adiposity but lacks the specificity required to quantify VAT separately from SCAT [15].

Despite the strengths and limitations of each method (Table 1), the integration of AI driven analytical techniques is revolutionizing VAT assessment by enhancing precision, reducing observer variability, and enabling large scale analysis of imaging data [1]. AI models trained on MRI and CT datasets can automate VAT and ectopic fat segmentation. This aims to reduce the time and expertise required for manual analysis [21]. For example, radiomics-based approaches using deep learning enhanced quantification of ectopic fat by extracting subvisual textural features related to fatty infiltration, seen in CT-quantification of liver fat [22]. Similarly, AI-enhanced ultrasound interpretation holds promise for improving the accuracy and reproducibility of VAT estimates, making the technique more accessible in primary care settings where other imaging modalities might not be available [23]. As AI continues to evolve, the fusion of multimodal imaging approaches is bound to provide more comprehensive and individualized assessments of VAT related cardiovascular risk [24].

## AI Techniques in VAT Quantification

**Fig. 1:**
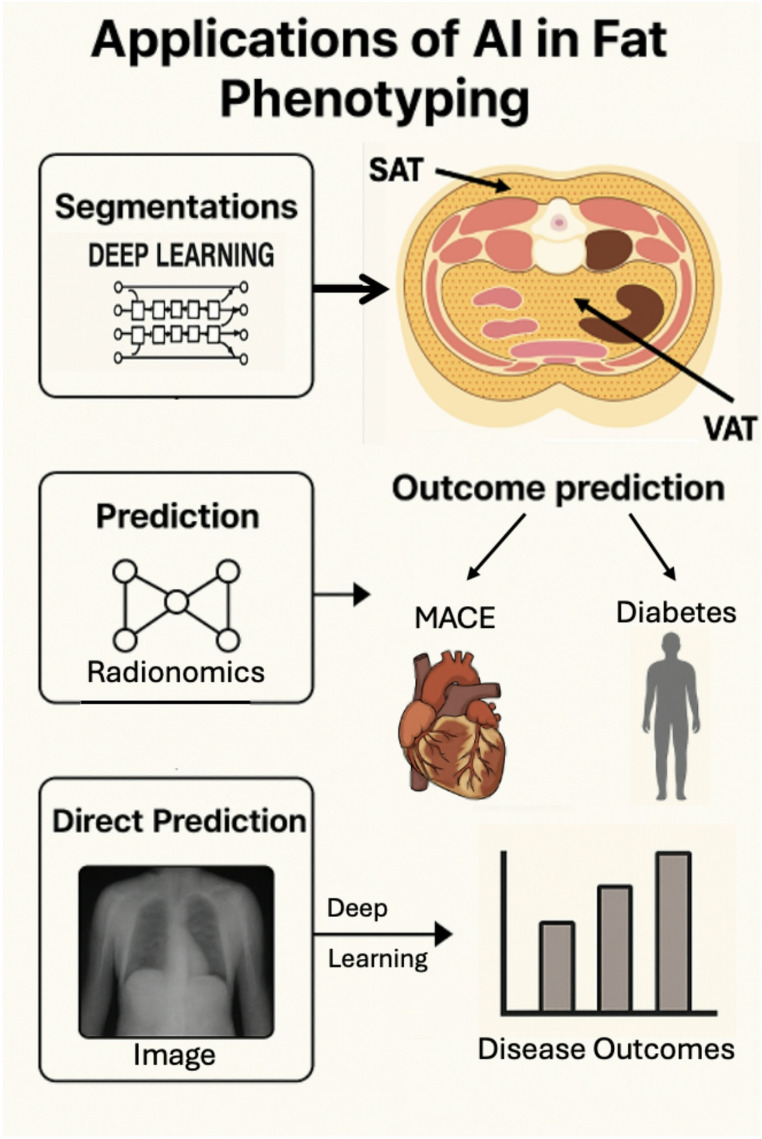
Applications of artificial intelligence in fat phenotyping. Deep learning (DL) models, such as U-Net, can be used to segment different visceral adipose tissue (VAT) depots. These segmentations enable radiomics feature extraction, which can then be input into machine learning (ML) models to predict clinical outcomes such as major adverse cardiovascular events (MACE) and diabetes. Alternatively, DL models can be applied directly to imaging data to predict disease outcomes without the intermediate step of feature extraction

Programs powered by AI provide an automated, precise, and efficient approach to VAT and ectopic fat imaging. These often work by reducing human error and enabling large scale analysis [24].By leveraging deep learning, machine learning, and radiomics, AI is set to revolutionize adipose tissue analysis, making it an invaluable tool in preventive cardiology and metabolic research [3, 25, 26] (Fig. 1).

### Deep Learning

Deep learning, a specialized and advanced subset of ML, leverages neural networks with multiple layers to process vast amounts of imaging data [1]. Convolutional Neural Networks (CNNs) have proven particularly effective in adipose tissue imaging due to their ability to detect spatial hierarchies in images [27]. CNNs are instrumental in processes such as image segmentation where different layers of adipose tissue are delineated. It has also been proven beneficial in feature extraction, which is described as the process which identifies relevant biomarkers from imaging data [27]. Prior to AI developments, traditional segmentation methods relied on manual annotation, which was time consuming and prone to inter-observer variability [27]. AI powered segmentation algorithms, however, work to automate this process, ultimately improving reproducibility and efficiency [27]. Studies have shown that CNN segmentation models achieve high Dice similarity coefficients, a measure of spatial overlap, when compared to expert manual segmentations [28]. Advanced deep learning models, including those using U-Net and ResNet architectures, have demonstrated superior accuracy in segmenting VAT and SCAT [29]. These advancements allow for automated image analysis, ultimately reducing manual labor and improving diagnostic consistency [30]. Furthermore, deep learning techniques enable the detection of minute and subtle changes in fat composition, aiding in early disease prediction and risk assessment for metabolic disorders [30].

Feature extraction is another crucial application of deep learning in adipose imaging [31]. AI models can analyze imaging data to identify complex features that may not be visible to the human eye [31–33]. For example, VAT and ectopic fat has differing morphology, texture, and density which are associated with metabolic and cardiovascular risks [32]. Radiomic features extracted from MRI or CT scans can provide insights into beyond traditional screening measures. These extracted features can then be used in predictive modeling to assess disease risk, monitor treatment response, and personalize patient management strategies [22, 33, 34].

### Integration of Multi-Modal Data

AI’s ability to integrate data from multiple sources including imaging modalities, clinical records, and genetic information has the potential to revolutionize the study of VAT and its relationship with CV risk factors [35]. Multi-modal learning techniques combine imaging data from MRI, CT, and ultrasound with patient information such as genetic predisposition, lifestyle factors, and metabolic markers [35]. This approach is much more comprehensive, and enhances risk stratification by allowing for a more nuanced understanding of the interplay between adipose tissue distribution and CVD [36]. Multimodal AI models can also predict patient outcomes by correlating imaging biomarkers with longitudinal health data, therefore offering a more personalized approach to preventive healthcare [36]. The integration of AI in multimodal data analysis also facilitates simultaneous monitoring of adipose tissue changes, which is critical in obesity management and vital for the timing of treatment interventions [36].

## Clinical Applications of AI in VAT and Ectopic Fat Assessment

### Automated VAT Measurement

AI algorithms, particularly deep learning models, have been developed to automate the segmentation and quantification of VAT from imaging modalities like CT and MRI. These automated methods work to address the limitations of manual assessments including time consumption and interobserver variability. For instance, a study involving over 9,000 asymptomatic adults demonstrated that AI-based measurements of abdominal adipose tissues, including VAT, were effective in predicting all-cause mortality, CVD, and diabetes [37]. Further advancements include the development of AI algorithms capable of segmenting adipose tissue compartments on CT and MRI images, facilitating comprehensive analysis of VAT and SAT [1, 38]. More examples of AI models include FatSegNet, which has been designed for quick and fully automated segmentation of abdominal adipose tissue on MRI. These models demonstrate high accuracy and reliability when studied [39].

One study proposed an automatic segmentation approach using deep CNNs for VAT, SCAT, and muscle at the L3 vertebra. This method achieved high accuracy and proved to surpass manual segmentation, especially in SCAT quantification [27]. In another study, deep learning was used to extract body composition data from CT scans, and found VAT to SCAT ratio to be an independent predictor of survival in lung cancer patients [40]. Other studies involving deep learning models include one that validated the DeepMedic CNN for precise body tissue segmentation and another one that developed a multi-resolution 3D U-Net for quantification [30, 41].

### Risk Stratification and Early Detection of Cardiovascular Disease

AI models have significantly advanced CVD risk stratification by integrating fat and tissue radiomics with clinical parameters, offering a more precise and comprehensive approach compared to traditional measures such as BMI and waist circumference [42]. Research indicates that visceral fat is superior to BMI and waist circumference for predicting cardiovascular risk, further underscoring the value of AI in risk assessment models by incorporating detailed VAT analyses [42–44]. Imaging techniques using AI facilitates the early detection of CVD by identifying subtle changes in VAT distribution and metabolic health markers that are often too minute to be detected [45]. Images from CT scans performed for calcium scoring can also be leveraged by AI to use deep learning techniques to segment epicardial adipose tissue (EAT) and engineered radiomics features of calcifications. When these features are combined, they are known as “calcium-omics” and “fat-omics”, and act with models of traditional risk factors to predict incident CVD [33].

AI derived measurements of EAT have been shown to predict major adverse cardiovascular events (MACE) with greater accuracy than traditional risk factors alone [45, 46]. These advancements are especially valuable for refining risk assessment models, enabling clinicians to incorporate detailed VAT analyses into their diagnostic and management plan [45]. Deep learning methodologies have significantly advanced the automated analysis of EAT, offering a more precise and efficient alternative to manual segmentation [47]. Recent studies have demonstrated that AI models can quantify EAT volume and attenuation from low-dose, ungated CT scans with expert-level accuracy, reducing variability and processing time [39, 52]. Furthermore, the integration of pericardial anatomical structures into deep learning algorithms has further enhanced the automatic detection and quantification of EAT [48]. These advancements allow clinicians to identify early atherosclerotic changes and alterations in adipose tissue related to inflammation which corelates with early diagnosis and preventive interventions for CVD [48].

Pericoronary adipose tissue (PCAT) has also emerged as an important imaging biomarker in cardiovascular risk assessment. Unlike epicardial adipose tissue, PCAT refers specifically to the fat immediately surrounding the coronary arteries, which reflects local vascular inflammation. Adverse PCAT phenotypes, such as higher attenuation values, have been shown to correlate with high-risk plaque features and independently predict major adverse cardiovascular events (MACE) [49–51]. AI has accelerated this field by enabling automated segmentation of PCAT and extraction of radiomic features, allowing “fat radiomics” to be integrated with coronary CT angiography for risk prediction [52]. By capturing vessel-specific inflammatory signatures, PCAT quantification complements VAT and EAT analyses, offering a more granular approach to cardiovascular risk stratification [53].

Body composition analyses driven by AI have been linked to important clinical outcomes such as higher rates of mortality in patients undergoing critical procedures like transcatheter aortic valve implantation [54] (TAVI). This further underscores the potential of AI in preoperative risk evaluation and shows how AI may be used to identify higher risk patients prior to surgery [54]. A novel obesity phenotyping approach using AI further demonstrates the potential of VAT assessment to revolutionize cardiovascular risk stratification, suggesting the need for broader research to establish standardized applications of these technologies [55]. In addition to risk assessment, AI has facilitated a deeper understanding of adipose tissue dynamics during aging and their implications for metabolic health [56]. One study employed machine learning to evaluate age related changes in fat distribution and was able to identify abdominal fat as a strong indicator of biological aging [56]. Other studies focus on refining segmentation methods to improve the accuracy of VAT measurement. These advancements have incorporated innovative techniques such as texture analysis and machine learning algorithms to more effectively identify metabolic risks [57–61].

## Challenges and Limitations

### Data Quality and Availability

Despite the rapid advancements in the application of AI to adipose imaging, there are several challenges that potentially hinder the widespread adoption of these technologies into clinical practice and research. One of the primary challenges in leveraging AI for adipose imaging is the lack of high quality and annotated datasets. ML models, particularly deep learning algorithms, require large amounts of labeled data to learn accurate patterns and make predictions. Advances in the development of foundation models, which can learn patterns within the data without explicit labeling can lessen the need for labeled data [62, 63]. Nevertheless, comprehensive and high quality datasets, particularly those covering diverse populations in terms of age, gender, ethnicity, and comorbidities, are often helpful in the development of robust models [64]. This issue of data scarcity is further exacerbated by the fact that the process of annotating data is time consuming and requires significant expertise [65].

### Interpretability of AI Models

The interpretability of models is another major limitation in the adoption of AI for adipose imaging. Deep learning (DL) models in particular operate as “black boxes,” meaning they often make predictions without providing clear explanations for their decision making processes [66]. This lack of transparency can be problematic in clinical settings because the inability to interpret AI models can lead to reluctance among clinicians. Understanding how AI models interpret specific patterns in adipose tissue, such as variations in visceral fat or adiposity distribution, is essential for ensuring that these tools are used correctly [67].

However, in the context of segmentation tasks, DL models offer a partial solution to this challenge [68]. Segmentation networks, such as U-Net and its variations, create an explicit, visualizable output that depicts certain anatomical structures including SAT and VAT [69]. These intermediate segmentation masks establish a way for the clinician to assess the model’s work, adjust borders if necessary, and retain some degree of interpretability in the workflow. Therefore, the DL-based segmentation can serve both as an analytic strategy, as well as a “processing modality” that interprets an input of raw image data to link it with a subsequent predictive model [70].

In fact, many prediction models based on VAT have a DL segmentation function as an initial step to create quantified fat metrics including VAT area, SAT area, and VAT/SAT ratio that can be used in the context of a statistical or machine learning model for cardiovascular risk prediction. The two-step process of separating the interpretable segmentation phase from the prediction phase leads to a fully transparent image analysis process while instituting less interpretable complex models for defining outcomes [71]. This tiered architecture could help address clinician concerns about “black box” algorithms when machine learning accepts an intermediary step that is tangible, reviewable, and coherent with the interpretation standards of traditional imaging [72].

### Regulatory and Ethical Considerations

The application of AI in clinical practice raises several regulatory and ethical issues [73]. As AI tools begin to be implemented in healthcare settings it is essential to ensure that they meet stringent safety and efficacy standards. Regulatory bodies such as the Food and Drug Administration (FDA) in the United States and the European Medicines Agency (EMA) must establish clear guidelines for the approval of AI based medical devices. These administrations must also ensure that these tools are thoroughly tested before being introduced into clinical practice. This includes not only ensuring the accuracy of AI models but also evaluating their ability to generalize across different patient populations and clinical environments [74]. Ethical concerns also emerge particularly around issues of fairness and equity since AI models are only as strong as the data they are trained on. The concern is that if these datasets are not representative of diverse populations there is a risk that AI applications may disproportionately benefit certain groups while neglecting others [75].

## Future Perspectives

### Enhanced Algorithms

One of the most promising directions for AI in adipose imaging is the continued advancement of algorithms, particularly deep learning models. As AI techniques continue to evolve we can expect significant improvements in the accuracy and efficiency of VAT and ectopic fat assessment [76]. The development of more sophisticated models will also contribute to reducing errors in segmentation by improving the delineation of fat tissue boundaries. These enhanced algorithms will be able to process larger and more complex datasets. Once established, these models can work to provide more precise assessments in diverse populations such as those with atypical fat distribution [77].

### Integration with Other Technologies

Another exciting development in the future of AI in adipose imaging is the potential integration with other technologies like wearable devices and telehealth platforms. The combination of AI with continuous monitoring systems could enable concurrent tracking of adipose tissue and cardiovascular health [78]. Smartwatches and fitness trackers collect valuable data on patient’s physical activity and metabolic function [79]. When incorporated with AI, algorithms could analyze this data alongside imaging results to monitor changes in adipose tissue over time [80]. This integration could pave the way for proactive interventions by allowing clinicians to detect early changes in adiposity or cardiovascular health while patients remain asymptomatic. For example, AI-powered monitoring systems could alert healthcare providers to shifts in visceral fat distribution or increases in fat mass, prompting early interventions or lifestyle modifications [81].

### Personalized Medicine

AI’s capacity to analyze large datasets, including clinical, imaging, and genetic data, holds immense potential for advancing personalized medicine. By integrating adipose imaging with other clinical parameters such as patient history, genetic predisposition, and lifestyle factors, AI models can help tailor treatment strategies for obesity and cardiovascular risk to individual patients [82]. AI can help identify subgroups of patients who may be at higher risk for developing CVD due to specific patterns of adiposity. For example, the ability of AI to detect and analyze small changes in visceral fat accumulation may help identify individuals who are at increased risk long before traditional clinical assessments would detect these risks [83].

## Conclusion

The integration of AI into adipose imaging represents a transformative advancement in both clinical practice and research. As VAT and ectopic fat is increasingly recognized for its strong association with metabolic syndrome and CVD, the ability to accurately and efficiently assess body fat distribution is essential. AI tools, especially those leveraging advanced machine learning and deep learning, offer promising solutions for automating image analysis, enhancing segmentation accuracy, and extracting clinically relevant features from complex imaging data. The integration of multi-modal data further enables a holistic and personalized approach to risk stratification and disease management. As AI technology evolves it will play an increasingly vital role in transforming adipose imaging, ultimately improving CVD prevention and management strategies.

**Table 1:** Comparison of imaging modalities for VAT quantification

Imaging Modality	Type	Advantages	Limitations	AI Integration
MRI	Direct	Excellent soft tissue contrast; no radiation; precise VAT/SCAT differentiation	High cost; time-consuming; limited availability	High—AI improves segmentation & analysis
CT	Direct	High resolution; rapid imaging; reliable VAT quantification	Ionizing radiation; not ideal for repeat use	High—used in training deep learning models
DEXA	Indirect	Widely available; low radiation; total body fat assessment	Limited VAT specificity; lower spatial resolution	Moderate—AI can enhance region-specific accuracy
Ultrasound (US)	Indirect	Cost-effective; non-invasive; portable	Operator-dependent; limited depth in obese individuals	Emerging—AI assists in standardizing estimates
Plethysmography	Indirect	Non-invasive; estimates body fat via volume analysis	Cannot distinguish VAT from other fat depots	Minimal—rarely used for VAT-specific assessment

*AI*: artificial intelligence; *CT*: computed tomography; *DEXA*: dual x-ray absorptiometry; *MRI*: magnetic resonance imaging; *SCAT*: subcutaneous adipose tissue; *VAT*: visceral adipose tissue.

## Data Availability

No datasets were generated or analysed during the current study.
